# Circulating Serum Trefoil Factor 3 (TFF3) Is Dramatically Increased in Chronic Kidney Disease

**DOI:** 10.1371/journal.pone.0080271

**Published:** 2013-11-25

**Authors:** Ting-yi Du, Hui-ming Luo, Hai-chun Qin, Fang Wang, Qing Wang, Yang Xiang, Yun Zhang

**Affiliations:** 1 Key Laboratory of Animal Models and Human Disease Mechanisms of the Chinese Academy of Sciences and Yunnan Province, Kunming Institute of Zoology, The Chinese Academy of Sciences, Kunming, Yunnan Province, China; 2 University of the Chinese Academy of Sciences, Beijing, China; 3 Department of Clinical Laboratory Medicine, the First People’s Hospital of Yunnan Province, Kunming, Yunnan Province, China; 4 Department of Nephrology, The First People’s Hospital of Yunnan Province, Kunming, Yunnan Province, China; 5 Department of Gastroenterology, The First People’s Hospital of Yunnan Province, Kunming, Yunnan Province, China; 6 Department of Gynecology, The First People’s Hospital of Yunnan Province,Kunming, Yunnan Province, China; Biomedical Research Foundation of the Academy of Athens, Greece

## Abstract

**Objectives:**

Trefoil factor 3 (TFF3) is a small peptide that plays an important role in mucosal protection, cell proliferation, and cell migration. The aberrant expression of TFF3 is correlated with gastrointestinal inflammation, solid tumors, and other clinical diseases. The objective of this study was to identify the distribution characteristics of serum TFF3 in common clinical diseases.

**Materials and Methods:**

A large prospective randomized study of 1,072 Chinese patients was performed using an enzyme-linked immunosorbent assay (ELISA) to examine the serum TFF3 concentrations in patients with different diseases. A matched case-control study was conducted on patients with chronic kidney disease (CKD) stages 1–5. Immunohistochemistry (IHC) was performed using renal tissues to determine the relationship between the severity of CKD and the serum and urine concentrations of TFF3 peptides.

**Results:**

The mean serum concentrations of TFF3 in patients with CKD, metastatic and secondary carcinoma (MC) and acute gastroenteritis (AG) (200.9 ng/ml, 95.7 ng/ml and 71.7 ng/ml, respectively) were significantly higher than those in patients with other common clinical diseases. A positive correlation tendency was observed between the serum TFF3 concentrations and the severity of CKD. The mean serum TFF3 values for CKD stages 1–5 were 23.6 ng/ml, 29.9 ng/ml, 54.9 ng/ml, 85.0 ng/ml and 176.6 ng/ml, respectively. The same trend was observed in the urine TFF3 concentrations and the CKD stages. The creatinine(Cr)-corrected concentrations of TFF3 in urine were 367.1 ng/mg·Cr, 910.6 ng/mg·Cr, 1,149.0 ng/mg·Cr, 1,610.0 ng/mg·Cr and 3,475.0 ng/mg·Cr for CKD stages 1–5, respectively. IHC revealed that TFF3 expression was concentrated in tubular epithelial cells.

**Conclusions:**

The influence of kidney injuries must be fully considered when performing clinical TFF3 research. Further studies on TFF3 in CKD will contribute to our understanding of its pathological roles and mechanisms in other diseases.

## Introduction

The human trefoil factor (TFF) family consists of three small peptide members. These peptides are resistant to thermal and enzymatic digestion mainly due to their structure, which consists of 1–2 trefoil domains [1]. TFFs are involved in the wound healing process [2], [3]. TFF3, previously known as intestinal trefoil factor (ITF), contains one trefoil domain with 59 amino acid residues and has a molecular weight of approximately 6.6 kDa (monomer) or 13 kDa (dimer) [4]. Many studies have suggested that TFF3 peptides perform important functions, such as wound healing, mucosal protection, cell proliferation and cell migration *in vivo* and *in vitro*.

The specific roles and mechanisms of TFFs remain unclear; however, sufficient evidence from both clinical and experimental studies has indicated that TFF3 is involved in the pathological processes of several human diseases, such as mucosal disorders and cancer [4], [5]. Additionally studies have demonstrated that the aberrant expression of TFF3 *in vivo* is correlated with inflammation of the gastrointestinal tract [6], [7] and a variety of solid tumors, including gastric cancer [8], [9], breast cancer [10], [11] and other cancers [12], [13], [14]. Further information that was obtained from clinical trials revealed the potential value of TFF3 as a biomarker of tumor metastasis and a therapeutic target for clinical applications [15], [16], [17], [18]. However, it has been generally accepted by all researchers that more randomized multi-center clinical trials are needed.

In this large-sample investigation chronic kidney disease (CKD) was associated with increased serum TFF3 concentrations. A further matched case-control study determined that the serum and urine concentrations of the TFF3 peptides were correlated with the stage of CKD severity and that these increased concentrations of TFF3 may be secreted from renal tubular epithelial cells in damaged kidneys.

## Materials and Methods

### Clinical sample collection

The sample collection was performed during two time periods. First, a total of 1,072 serum samples (200 µl per case) were randomly collected from July 2011 to September 2011 to compare the serum TFF3 levels among patients with common clinical diseases. These samples included 50 healthy samples from a control group that were collected during routine health examinations within the same time period. To determine the relationship between the TFF3 concentrations (serum and urine) and the severity of CKD, 150 serum and urine samples were collected from April 2012 to July 2012. These samples were divided into 5 matched groups according to the stage of CKD (1–5), which was defined by the Practice Guidelines for Chronic Kidney Disease [19]. Each group contained 30 sample pairs from CKD patients (gender-balanced), and the equation that was used to estimate the glomerular filtration rate (GFR) was modified for Chinese patients with CKD [20]. Kidney tissue specimens were simultaneously collected in the second time period for IHC analysis. The samples were obtained by percutaneous renal biopsy from recruited patients who qualified for and consented to this invasive detection method [21], [22].

All of the samples were collected at the First People’s Hospital of Yunnan Province. The serum and urine samples were stored at –80°C until use, and the kidney tissue specimens were routinely fixed in 10% formalin and embedded in paraffin.

### Ethics statement

The Ethics Committee of the Kunming Institute of Zoology, the Chinese Academy of Sciences approved the study, and the Ethics Board of Clinical Medicine from the First People’s Hospital of Yunnan Province, China approved the sample collection. Written informed consent was obtained from the donor or the next of kin to use the samples in this study.

### Serum and urine TFF3 immunoassay

According to a previously reported protocol [15], an in-house sandwich enzyme-linked immunosorbent assay (ELISA) was established to measure the concentrations of TFF3 in the serum and urine specimens. To develop the ELISA, a mouse monoclonal anti-TFF3 antibody (catalog No: H00007033-M01, clone 3D9; Abnova Corporation, Taipei, Taiwan) was used as the capture antibody at 2 µg/ml, and a rabbit polyclonal anti-TFF3 antibody, ITF FL-80 (catalog No: sc-28927; Santa Cruz Biotechnology, Inc., Dallas, Texas, USA 75220,), was used as the detection antibody at 0.4 µg/ml. A horseradish peroxidase (HRP)-conjugated goat anti-rabbit antibody was used at a dilution of 1:5,000 (catalog No: 074-1516; KPL, Inc., 910 Clopper Road, Gaithersburg, Maryland, USA), and a TMP HRP color development solution for ELISA (catalog No: P0209; Beyotime Institute of Biotechnology, Haimen, Jiangsu, China) was used to generate the enzyme-catalyzed color reaction. Absorbance was measured at 450 nm using an automatic plate reader (ELx800 Absorbance Microplate Reader, BioTek Instruments Inc., Highland Park, Winooski, VT, USA 05404).

The full-length recombinant TFF3 protein (catalog No: H00007033-P01, Abnova Corporation, Taipei, Taiwan) was diluted in assay buffer (PBS 1% w/v BSA, 0.05% Tween 20) to obtain a standard curve, and the assay buffer alone was used as a zero calibrator. For the clinical sample measurements, the serum samples were diluted at least 1:5, and the urine samples were diluted 1:50. Each sample was examined at least in duplicate.

### Immunohistochemistry of kidney tissue

Immunostaining of 23 formalin-fixed, paraffin-embedded kidney tissue samples was performed as previously described [23], [24] using the mouse monoclonal anti-TFF3 antibody (catalog No:H00007033-M01, clone 3D9) at 12.5 µg/ml and the HRP-conjugated goat-anti-mouse antibody at 1:5,000 (catalog No: sc-2005; Santa Cruz Biotechnology, Inc., Dallas, Texas, USA). Three independent pathologists blindly examined the slides.

### Statistical analysis

Statistical analyses were conducted using SPSS software (Statistical Package for the Social Sciences) version 16.0 (SPSS Inc., Chicago, IL, USA). For data processing, the values that were below the standard curves were scored as the lowest detectable value of each assay, and the extreme high values within each group were excluded to emphasize the central tendency of the data distributions. The differences in the TFF3 concentrations among the groups were compared using one-way analysis of variance (ANOVA) (Student-Newman-Keuls q test), the correlation between urine TFF3 concentration and proteinuria was calculated using the Pearson correlation analysis, and the statistical threshold was set at 0.05 (two-sided). The figures were generated using GraphPad Prism software, version 5.01 (GraphPad Software, Inc., Avenida de la Playa, La Jolla, CA, USA).

## Results

The sensitivity of the ELISA in this study was ≤0.78 ng/ml, and the linear range was 1.56–100 ng/ml (r^2^ = 0.998). Over a concentration range of 0–500 ng/ml, no hook effects or cross-reactivity with recombinant TFF1 and TFF2 proteins were observed. The results of the spike-and-recovery assessment (the spiked TFF3 concentrations were 10 ng/ml, 50 ng/ml and 90 ng/ml) indicated that the recovery rate of the ELISA was influenced by the dilution. For the serum samples that were diluted 1:5, the recovery range was 87.8–89.6%, and for the urine samples that were diluted 1:50, the relevant recovery range was 100.4–125.5%. The evaluation of the method reliability confirmed that the intra-assay and inter-assay coefficients of variation (CVs) were 6.0% and 14.8%, respectively.

A total of 1,500 in-patient cases were randomly chosen for serum collection. Overall, 357 cases were excluded from the study because they met the exclusion criteria ( the patients were not within the age range of the study or had an inconclusive diagnosis or too few cases were available to compose a group). Additionally, 71 cases with extremely high values that were distributed among the groups were excluded. Therefore, a total of 1,072 cases were divided into 30 groups (Table 1) for further analysis. The proportions of diseases in Table 1 may represent the distribution of common diseases in the Yunnan Province, and the original data are presented in Dataset S1.

**Table 1:** Serum TFF3 concentrations in groups of patients with common clinical diseases.^a^

Patient group ( group label )	Cases (Male cases)	Age( years ) b	Serum TFF3 ( ng/ml ) b
			
**Normal control ( NC )**	**50(21)**	**54.5 (20 – 77)**	**17.8 (11.6 – 25.1)**
**Viral hepatitis ( VH )**	**14(10)**	**46.5 (16 – 75)**	**23.7 (12.6 – 46.6)**
**Bacterial infections of**			
**respiratory system ( BIR )**	**42(26)**	**74.5 (15 – 85)**	**34.0 (12.5 – 84.6)**
**urinary tract and pelvic cavity ( BIUP )**	**34(3)**	**32.0 (23 – 81)**	**23.9 (16.8 – 35.6)**
**Tuberculosis infection ( TB )**	**33(19)**	**49.0 (18 – 79)**	**23.1 (10.3 –41.6)**
**Digestive system diseases**			
**Acute gastroenteritis ( AG )**	**11(6)**	**70.0 (34 – 81)**	**63.5 (15.5 – 155.4)**
**Gastrointestinal ulcer/chronic inflamation ( GUCI )**	**64(29)**	**57.5 (29 – 82)**	**28.4 (7.8 – 64.5)**
**Hepatobiliary disease ( HBD )**	**89(37)**	**57.0 (26 – 84)**	**24.55 (11.0 – 51.8)**
**Pancreatic disease ( PD )**	**16(8)**	**45.0 (22 – 67)**	**20.9 (12.2 – 35.2)**
**Cardiac-cerebral vascular diseases**			
**Essential hypertension ( EH )**	**71(45)**	**63.0 (32 – 85)**	**31.0 (11.1 – 68.4)**
**Cerebrovascular accident ( CVI )**	**51(34)**	**69.0 (33 – 84)**	**31.9 (12.2 – 63.1)**
**Ischemic cardiomyopathy ( IC )**	**29(24)**	**63.0 (37 – 84)**	**27.1 (11.1 – 43.8)**
**Diabetes mellitus and its complications ( DMC )**	**47(28)**	**71.0 (32 – 85)**	**35.5 (12.2 – 84.8)**
**Thyroid disorders ( TD )**	**9(4)**	**40.0 (16 – 58)**	**21.2 (19.0 – 27.6)**
**Renal diseases**			
**nephritis ( NEPH )**	**19(7)**	**34.0 (21 – 73)**	**24.6 (16.5 – 40.0)**
**chronic kidney disease(CKD)**	**40(26)**	**56.5 (26 – 83)**	**192.4 (64.2 – 395.3)**
**lithiasis, immune-related nephrosis and others ( LIN )**	**45(23)**	**46.0 (14 – 83)**	**33.6 (9.6 – 126.3)**
**Autoimmune diseases ( AD )**	**28(5)**	**43.5 (14 – 67)**	**25.6 (17.7 – 48.2)**
**Hyperplasia and benign tumour of**			
**respiratory system ( HBTR )**	**20(11)**	**42.5 (18 – 80)**	**24.1 (20.3 – 35.0)**
**digestive system ( HBTD )**	**35(24)**	**45.0 (19 – 77)**	**27.0 (15.2 – 64.6)**
**prostate (HBTP )**	**22(22)**	**73.0 (56 – 85)**	**29.9 (10.5 – 54.1)**
**breast ( HBTB )**	**44(0)**	**41.0 (17 – 70)**	**20.6 (8.9 – 36.3)**
**gynecology (HBTG )**	**68(0)**	**35.0 (14 – 81)**	**21.9 (11.1 – 35.8)**
**thyroid and others ( HBTT )**	**34(12)**	**48.0 (16 – 85)**	**24.7 (11.2 – 44.1)**
**Malignant tumour of**			
**respiratory system ( MTR )**	**22(16)**	**56.5 (33 – 78)**	**28.2 (17.5 –42.5)**
**digestive system ( MTD )**	**54(36)**	**59.0 (32 – 80)**	**33.0 (12.3 – 63.7)**
**breast ( MTB )**	**18(0)**	**50.0 (35 – 69)**	**26.5 (9.4 – 40.8)**
**urogenital system ( MTU )**	**17(5)**	**61.0 (25 – 79)**	**24.4 (14.7 – 61.9)**
**metastatic and secondary carcinoma ( MC )**	**23(12)**	**70.0 (41 – 81)**	**62.5 (22.0 –195.2)**
**others ( MTO )**	**23(9)**	**46.0 (16 – 73)**	**24.5 (11.9 – 37.1)**
**Total**	**1072(502)**	**55.0 (14 – 85)**	

a The original data are presented in Dataset S1.

b The data were showed as the Median (Range) in the table.

The serum concentrations of TFF3 in each common clinical disease group are presented in Table 1. There were no significant differences between the groups with these common clinical diseases based on the one-way ANOVA analysis (Student-Newman-Keuls q test). However, significant differences were identified between the CKD group and the other groups. Moreover, the statistical characteristics of the metastatic and secondary carcinoma (MC) group and the acute gastroenteritis (AG) group were similar to those of the CKD group. The statistical efficiency of this study was influenced by the sample collection and the statistical analysis methods; however, the prime disease factors that were associated with significantly increased serum TFF3 concentrations were, in order of significance, CKD, MC and AG.

**Figure 1:**
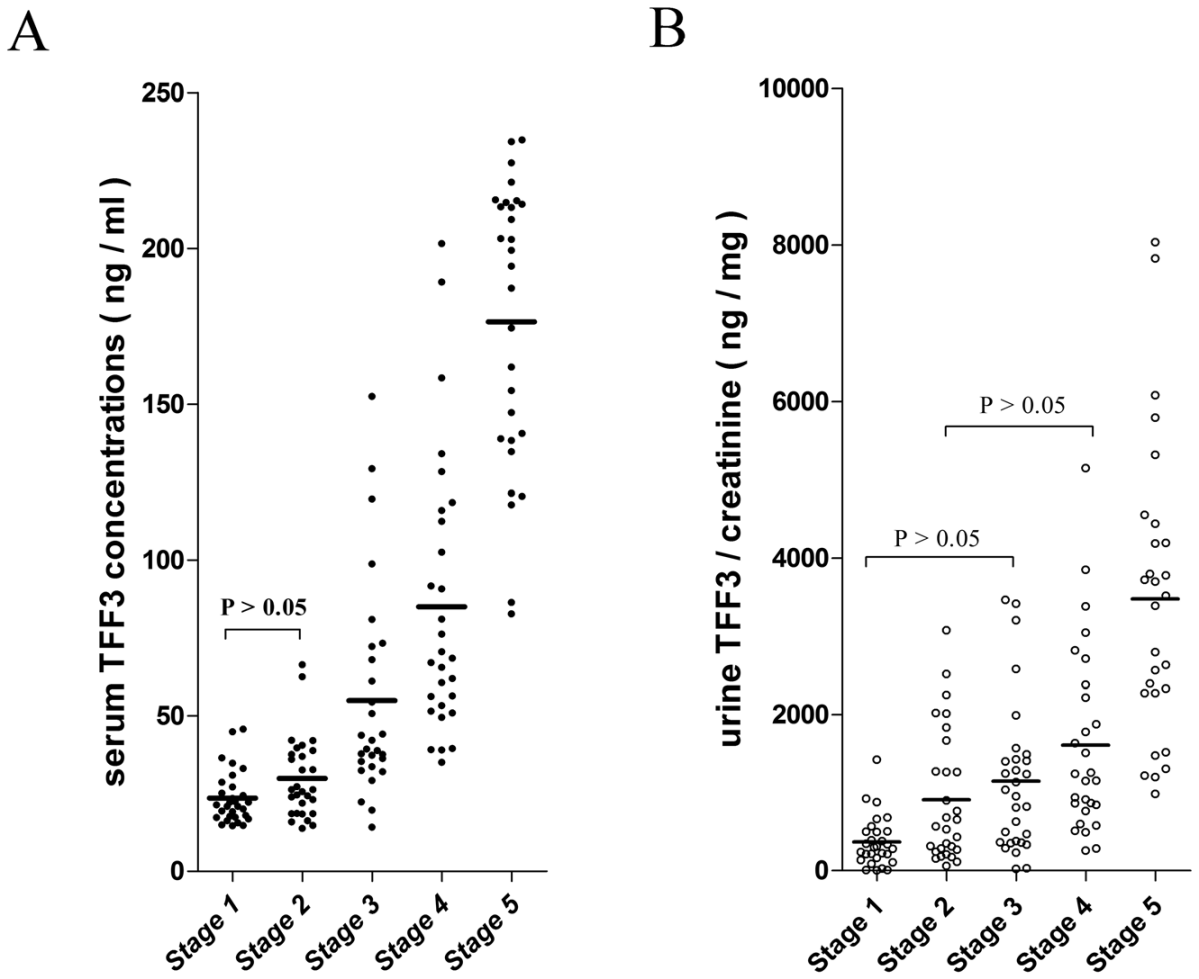
A scatter plot of TFF3 in 5 matched patient groups with different CKD stages. (A) The TFF3 concentrations in sera. (B) The TFF3 concentrations in urine. The mean values are shown as "—" in each column.

**Figure 2:**
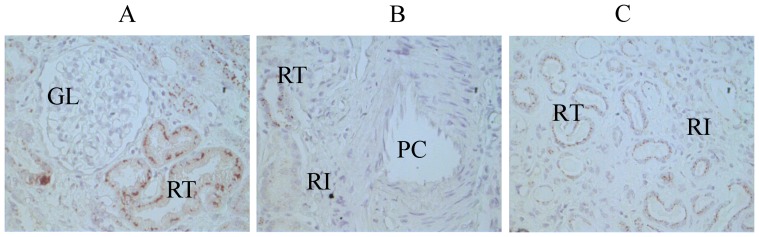
TFF3 expression according to immunohistochemical staining of kidney tissue from a patient with stage 2 CKD. (A) the renal cortex, (B) a renal peritubular capillary, and (C) the renal medulla. GL: glomeruli, RT: renal tubule, RI: renal interstitium, PC: peritubular capillary. The samples were examined using S–P immunohistochemical staining. The pictures are shown at 400x magnification.

The clinical and demographic data from the CKD sub-groups (Dataset S2) are briefly summarized in Table 2. The results of the matched case-control study are presented in Figure 1. The serum and urine concentrations of TFF3 increased with the severity of CKD. The Pearson’s correlation coefficient between urine TFF3 concentration and urine total protein ( both creatinine-corrected ) was 0.24 ( p  =  0.006 ). Furthermore, the IHC analysis of 23 tissue specimens that were collected by renal biopsy (11 cases of stage 1 CKD, 8 cases of stage 2 CKD and 4 cases of stage 3 CKD) indicated that the aberrant expression of TFF3 was localized to renal tubular epithelial cells. No clearly positive reactions for TFF3 were observed in the renal glomeruli, the peritubular capillaries or the renal interstitium in the biopsy specimens. The representative IHC results are showed in Figure 2

**Table 2:** A summary of the test results related to renal function in the CKD sub-groups.^a^

CKD stage	Cases	Age	GFR	SCr	BUN	UTP
		(years)	(ml/min/1.73m2)	(umol/L)	(mmol/L)	(mg/24hr)
**1**	**30**	**37.5 (17 – 68)**	**137.3 (90.2 – 362.0)**	**58.0 (32.0 – 86.0)**	**4.55 (1.2 –7.9)**	**808 (97 – 3710)**
**2**	**30**	**49.5 (20 – 76)**	**77.9 (60.1 – 88.7)**	**90.0 (72.0 – 129.0)**	**6.35 (3.3 – 14.1)**	**1579 (67 – 9540)**
**3**	**30**	**55.0 (16 – 78)**	**45.9 (30.4 – 59.7)**	**133.1 (96.5 – 206.0)**	**8.55 (4.1 – 16.0)**	**1399 (67 – 6492)**
**4**	**30**	**53.0 (19 – 83)**	**21.3 (15.6 – 28.9)**	**256.0 (188.0 –354.0)**	**11.65 (6.0 – 20.1)**	**1528 (84 – 6526)**
**5**	**30**	**48.0 (24 – 80)**	**6.1 (2.4 – 14.8)**	**639.3 (325.6 – 1812.0)**	**23.1 (7.6 – 81.6)**	**2145 (133 – 7695)**

a Additional clinical material is presented in Dataset S2. The data were showed as the Median (Range) in the table. GFR: glomerular filtration rate, SCr: serum creatinine, BUN: blood urea nitrogen, UTP: Urine total protein.

## Discussion

TFFs are multi-functional peptides that are synthesized and secreted by mucin-secreting epithelial cells in amphibians and vertebrates. TFFs are highly conserved during evolution, and the conserved amino acid residues are necessary for their molecular structure and biological functions [25]. Many comprehensive studies have accessed their unique biochemical properties and multiple functional effects since the discovery of the pancreatic spasmolytic polypeptide (PSP) 30 years ago [26].

Mammalian TFF3 was identified in 1991, and its nomenclature was standardized in 1997 [27], [28]. Previous studies have confirmed that under normal conditions, TFF3 is most abundantly expressed in the gastrointestinal tract. A lower level of expression can be detected in tissue that contains mucus-secreting cells, which suggests that their functional effects may be associated with those of mucins [1], [4]. Therefore, research has been concentrated on the aberrant expression of TFF3 in different pathophysiological processes, particularly the pathological roles of TFF3 and the mechanisms of abnormal TFF3 expression. Clinical studies have indicated that TFF3, both the mRNA and the peptide may be a valuable biomarker candidate[14], [15], [16], [17]. To validate its clinical use, large randomized trials with validated clinical detection methods and samples are essential [29], [30], [31], [32].

The TFF3-ELISA assay has been used in many laboratory and clinical studies over the last decade. It’s difficult to compare the results from different laboratories because of methodological discrepancies; however, improvements have been made according to a previous report [33]. Moreover, physiological variations have only been partially investigated [34]. Nonetheless, studies on the diagnostic potential of serum TFF3 have gradually concentrated on its clinical use in the diagnosis and treatment of malignant tumors.

The results of the random sampling survey in this study clearly demonstrated that the serum TFF3 concentrations were much higher in the CKD group than in the normal control (NC) group and the MC group (mean values: 200.9 ng/ml, 18.0 ng/ml and 95.7 ng/ml, respectively). Moreover, the data indicated that the serum TFF3 levels were higher in patients with CKD than in patients with any other common clinical disease, which suggests that kidney injury may be an unavoidable factor to consider in future studies.

As shown in Figure 1, the mean serum TFF3 values for CKD stages 1–5 were 23.6 ng/ml, 29.9 ng/ml, 54.9 ng/ml, 85.0 ng/ml and 176.6 ng/ml, respectively. The serum TFF3 concentrations increased with the severity of CKD. The difference between the values at stages 1 and 2 was small compared with the changes that were observed in the values at other stages of CKD; however, this difference was statistically significant according to an independent sample *t*-test. The same trend was observed in the urine TFF3 concentrations and the severity of CKD. The creatinine-corrected concentrations of TFF3 in urine were 367.1 ng/mg·Cr, 910.6 ng/mg·Cr, 1,149.0 ng/mg·Cr, 1,610.0 ng/mg·Cr and 3,475.0 ng/mg·Cr for the 5 CKD stages, respectively. This positive correlation tendency and the IHC results indicated that TFF3 may play an important pathological role in the disease progression of CKD, particularly in the pathological process of renal tubular lesions.

Too few renal biopsy specimens were available in this study; therefore a more constructive conclusion could not be reached. However, the lack of a correlation between the levels of TFF3 and proteinuria (r  =  0.24) in this study strongly suggests that glomerular injury is not associated with urinary TFF3 excretion. Additionally, Rinnert et al. used RT-PCR to demonstrate that TFF3 expression was detectable in all portions of the urinary tract with peaks in the renal medulla and the urethra [35], and the results of the immunofluorescence analysis in this study demonstrated that TFF3 expression was localized to tubular cells in the renal cortex. Therefore, renal tubular lesions may lead to an increase in TFF3 in sera and urine during the progression of CKD. However, to test this hypothesis, a comparison of TFF3 expression levels under normal conditions with those in CKD is needed in future studies. Additionally, measuring markers of tubular injury, such as neutrophil gelatinase-associated lipocalin (NGAL) and kidney injury molecule-1 (KIM-1), would help elucidate the relationship between renal tubular injury and TFF3.

Several reports have focused on the pathological roles and practical applications of TFFs in urological diseases. TFF1 is a novel and potent CaOx crystal growth inhibitor with a potential pathophysiological role in nephrolithiasis [36]. TFF2 has been demonstrated to be the predominant TFF peptide excreted in urine, and significantly increased urine TFF2 levels, together with occasionally increased TFF3 levels have been observed in patients suffering from nephrolithiasis. Additionally, TFF3 is the predominant TFF peptide that is synthesized in urinary tract epithelia [35]. In rats, TFF3 protein levels were reduced in response to acute renal tubular injury, and urinary TFF3 and albumin enabled a more sensitive and robust diagnosis of acute renal tubular injury than traditional biomarkers [37]. A case-control study that was performed over 8.6 years found that higher urinary TFF3 levels were associated with CKD, which suggests that TFF3 may be a useful marker of future risk of CKD [38].

The pathological roles of TFF3 in urological diseases remain ill defined. Based on its biological effects, TFF3 may play key roles in regeneration and restitution processes [35] and in the ongoing repair of kidney damage [38]. An experimental study in 2003 implicated TFF3 as a proangiogenic factor because the aberrant expression of TFF3 was associated with the formation of new blood vessels during normal and pathophysiological processes that involved neovascularization [39].

It is well known that renal interstitial fibrosis (RIF), which is mediated by the signal channel, is a common pathway that leads to end-stage renal disease (ESRD) during most types of clinical CKD, and that the degree of RIF is more closely correlated with renal function than glomerular sclerosis. Several studies have demonstrated that the epithelial-to-mesenchymal transition (EMT) plays a key role in RIF [40], [41], [42] and in the chronic fibrosis of many organs [43], [44]. Furthermore, EMT was reported to be closely correlated with the invasion and metastasis of malignant epithelial tumors [45], [46]. Considering the established data and the results of this study, which demonstrated that the expression levels of TFF3 in CKD, MC and AG differed from those in other disease groups, we hypothesized that TFF3 participates in the EMT process. EMT is a general pathway that is involved in the pathological processes of CKD, MC and other diseases. This hypothesis may explain the aberrant expression of TFF3 that was observed in this study; however, confirmatory evidence that epithelial cells are an important source of myofibroblasts *in vivo* is lacking. Notably, one study provided definitive lineage tracing evidence that kidney epithelial cells do not become myofibroblasts *in vivo* [47]. Therefore, more detailed and extensive studies will be needed to clarify the pathophysiological role of TFF3 in CKD.

In conclusion, the results from this study demonstrate that among all common clinical diseases, CKD is the foremost disease that is associated with a dramatic increase in serum and urine TFF3 levels. The increased TFF3 peptide levels may result from TFF3 secretion by injured renal tubular epithelial cells. We hypothesize that TFF3 is

involved in the pathological process of EMT. If corroborated by other studies, this finding could accelerate pathomechanism research and the development of TFF3 for clinical diagnostic practices.

## Supporting Information

Dataset S1 **The original data of 1072 patients analysed in the study.**
(XLS)

Dataset S2 **The excerptions of medical records and examination results of 150 patients used in the CKD cohort study.**
(XLS)
